# Automatic and Real‐Time Surgeon's Gazing Point Detection From Surgical Videos Using Machine Learning and Mathematical Algorithm

**DOI:** 10.1002/jhbp.70052

**Published:** 2025-12-19

**Authors:** Shu Sasaki, Kenji Karako, Kyoji Ito, Yuichiro Mihara, Maho Takayama, Ryo Oikawa, Takeshi Takamoto, Nobuhisa Akamatsu, Yoshikuni Kawaguchi, Kiyoshi Hasegawa

**Affiliations:** ^1^ Hepato‐Biliary‐Pancreatic Surgery Division, Department of Surgery, Graduate School of Medicine The University of Tokyo Tokyo Japan; ^2^ National Center for Global Health and Medicine, Department of Hepatobiliary and Pancreatic Surgery Japan Institute for Health Security Tokyo Japan

**Keywords:** artificial intelligence, deep learning, pancreaticoduodenectomy, video recording

## Abstract

**Background:**

Application of artificial intelligence (AI) in intraoperative imaging has been expanding rapidly. The surgeon's gazing point indicates the exact site of surgical procedures and concentrates critical information for AI applications. This study aimed to develop a machine learning‐based system to automatically detect the surgeon's gazing point from surgical video data.

**Methods:**

Surgical instruments were detected using a deep‐learning model applied to images extracted from pancreaticoduodenectomy videos. Gazing points were estimated through a mathematical algorithm based on the axes and intersections of detected instruments, and time‐averaging was applied to enhance stability in real‐time analysis. After validation using pancreaticoduodenectomy cases, the system was subsequently applied to extended cholecystectomy and distal pancreatectomy cases to evaluate its applicability to other procedures.

**Results:**

Surgical instrument detection yielded AP50 of 60.5%. Gaze points detection achieved accuracies of 82.7% and 93.9% within 216‐ and 324‐pixel radii (9.42% and 21.2% of a 1440 × 1080 screen) in pancreaticoduodenectomy. When applied to extended cholecystectomy and pancreaticoduodenectomy distal pancreatectomy, our system demonstrated comparable performance, with an accuracy of 85.5% within the 324‐pixel radius. Time averaging improved accuracy, particularly with a 5‐s average.

**Conclusions:**

Our system successfully detected the surgeon's gaze point across procedures, suggesting potential utility in future AI‐assisted surgery.

## Graphical abstract

Sasaki and colleagues developed an automatic real‐time system to detect the surgeon's gazing point from surgical videos using a machine‐learning model and mathematical algorithm. Trained and validated on pancreaticoduodenectomy cases with high accuracy, the system showed comparable performance in distal pancreatectomy and extended cholecystectomy, highlighting its potential for future AI‐assisted surgery.

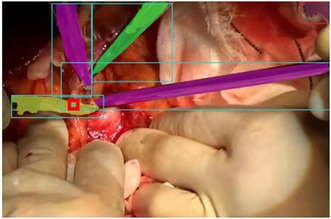

## 1 Introduction

The use of artificial intelligence (AI) in medicine has advanced rapidly in recent years, with diverse applications in genomic medicine [1], image diagnosis [2], and drug discovery [3]. AI‐assisted identification of surgical instruments and anatomical landmarks in minimally invasive surgery has been reported in intraoperative surgical imaging [4, 5, 6]. The use of AI for intraoperative surgical imaging is expected to expand in the future, including automated endoscopic camera control and robot‐assisted surgery.

The gazing point indicates the exact site where the surgeon focuses and the surgical procedure is performed. Moreover, important surgical information is concentrated around the gazing point. Therefore, in the clinical application of AI‐related technologies in surgery, the gazing point serves as the focal point for information collection and technology implementation. However, studies on AI‐based automatic detection of gaze points from surgical videos and images remain lacking. Moreover, the complexity of surgical procedures, scenes, and techniques poses challenges for AI to directly detect the gazing point.

The study aimed to develop a method to detect the gazing point from images extracted from surgical videos using machine learning and mathematical algorithms. Furthermore, we also applied this method to surgical videos and developed a system to automatically detect gazing points in real time. Automatic detection of intraoperative gazing points enables automated intraoperative videography, facilitates information sharing in the operating room, enhances patient safety, and provides materials for research and education.

## 2 Methods

The development of deep learning has enabled methods such as You Only Look Once [7] and Faster Regional Convolutional Neural Network (R‐CNN) [8] to detect objects in videos and images. These methods achieve object detection by labeling regions on the image and training a model on a large dataset of labeled images using a multilayered convolutional neural network specialized for image analysis. Each neural network contains numerous parameters. Therefore, the output in the learning step is compared with the correct data and optimized several times by varying the parameters to minimize the difference. However, the gazing point is not a specific object but a relative position that varies depending on the surgical situation. Therefore, directly detecting the gazing point using machine learning, as in object detection, is difficult. In this study, we developed a method to detect the gazing point relative to surgical instruments extracted through object detection. The general steps were as follows: detection of the surgical instruments, estimation of the surgical instrument axis, and calculation to detect the gazing point.

### 2.1 Patients and Images

Eight high‐quality videos were selected from 92 pancreaticoduodenectomies performed at The University of Tokyo Hospital between 2020 and 2021. Of the eight videos, three were used for training the machine learning model for surgical instruments, two were used for validating the instrument detection, and three were used for testing the entire system for gazing point detection. Approximately 100 images were manually captured from each of the five videos for surgical instrument detection, creating a diverse dataset covering various viewpoints and surgical procedures to ensure intrapatient diversity. Furthermore, a 60‐s video was extracted, and 60 images were automatically captured once per second from each of the three videos used to validate the entire system for detecting the gazing point. To evaluate the generalizability of our system to other procedures, 2 cases of open extended cholecystectomy and 4 cases of open distal pancreatectomy performed between 2024 and 2025 were selected. Using the same method as for PD, a 60‐s video was extracted, and 60 images were automatically captured once per second from each of 6 videos. This study was approved by the Research Ethics Committee of the Faculty of Medicine at the University of Tokyo (2021282NI‐(2)). Participants had the option to opt out and refuse consent.

### 2.2 Image Annotations and Machine Training for the Detection of Surgical Instruments

**FIGURE 1:**
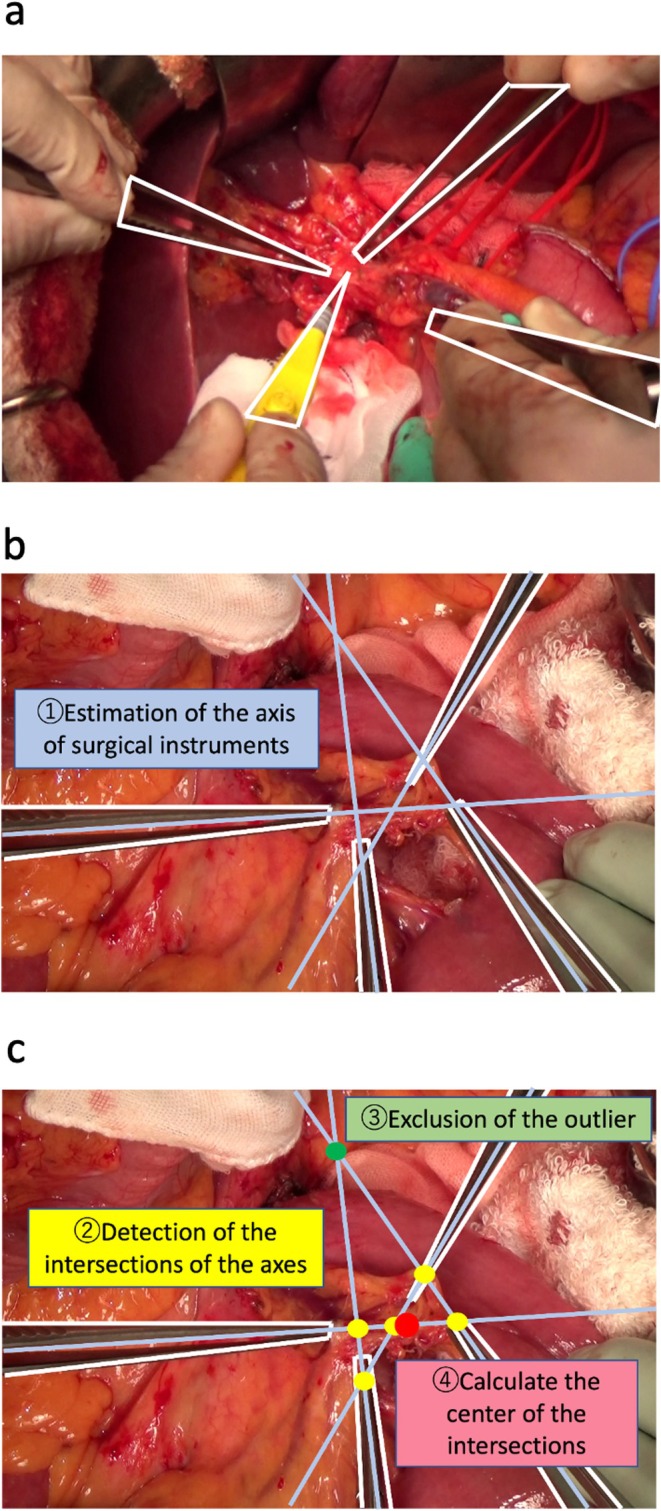
(a) Representative annotation image of the training dataset. Forceps and electrical scalpels are encircled with a white line and identified as surgical instruments. (b) Mathematical calculation of the axis of surgical instruments from the detected surgical instruments. Blue lines represent the calculated axes. (c) Calculation of the center of the gravity of the intersections of the axes after excluding outliers. Yellow dots are the included intersections, and green dots are the excluded intersections to calculate the center of the gravity of the intersections (red dot).

The images were manually annotated with all the surgical instruments, such as forceps and energy devices (Figure 1a), using Labelme software [9]. Two experienced hepatopancreatobiliary surgeons (S.S. and Y.M.) performed the labeling. Mask R‐CNN [10] is a well‐known deep‐learning algorithm for image detection. Therefore, we used MaskRCNN_ResNet50_FPN_Weights.COCO_V1, which is pre‐trained on common objects, to detect the surgical instruments, and PyTorch (Meta AI, New York, USA) was used for implementation.

Optimization of instrument detection requires iterative model training with correct data. The training procedure was as follows: First, the image of the training dataset was input into the model to predict the object coordinates and labels. Second, the error between the correct and predicted outputs was calculated. Third, the parameters were optimized using the “Adam” method [11], a common approach used in deep learning, to minimize errors. Adam is a gradient descent algorithm with adaptive momentum that computes adaptive learning rates for each neural network parameter individually. These processes are called “epoch.” The model was trained 50 times, and Adam was used to modify the mask R‐CNN parameters after each training.

### 2.3 Mathematical Algorithm to Estimate the Gazing Points

Our algorithm for estimating the gazing points was based on the hypothesis that the surgeon's gazing point approximates the intersection of the instrument axes. Our algorithm comprised the four steps: Step 1: Estimation of the surgical instrument axes from the detected instruments (Figure 1b); Step 2: Detection of the intersection of instrument axes as potential gazing points. Step 3: Exclusion of the outlier intersections. Step 4: Calculation of the center of gravity among the intersections as the gazing point (Figure 1c). The details of the algorithm are as follows:

- Step 1: Estimation of the surgical instrument axes from the detected instruments
  The axes of the surgical instruments were calculated using the detection area information. From the detected area of the target instrument, a mathematical algorithm was used to calculate the center of the instrument and the ends of the target instrument. Subsequently, the line connecting these points was estimated as the axis of the instrument. Details are presented in Figure S1.
- Step 2: Detection of the intersections of the instrument axes as the potential gazing points
  The axes of all the surgical instruments in the image were calculated, and the intersections of each axis were considered potential gazing points.
- Step 3: Exclusion of the outlier intersections
  Intersections between near‐parallel axes may deviate significantly from the actual gazing point and must be excluded. Exclusion of the outlier intersections was performed using “LocalOutlierFactor,” [12] implemented in scikit‐learn [13].
- Step 4: Calculation of the center of the gravity among the intersections as the gazing point
  The center of gravity of the remaining intersections, after outlier exclusion, was set as the estimated gaze point. The X coordinate was determined as the average of all X coordinates of the intersections, and the Y coordinate was calculated in a similar manner.

### 2.4 Time Averaging

The proposed system estimated the gaze point from still images. When this system was applied to the video, the gazing point was estimated for each frame (0.25 s). To avoid excessive fluctuations, a time‐averaging method was applied. Outliers were excluded as mentioned previously using “LocalOutlierFactor.” [12] We applied three different time averages: no time averaging (Method 1), time averaging every second (Method 2), and time averaging every 5 s (Method 3) and compared the accuracy of gazing point estimation.

### 2.5 Accuracy Determination

**FIGURE 2:**
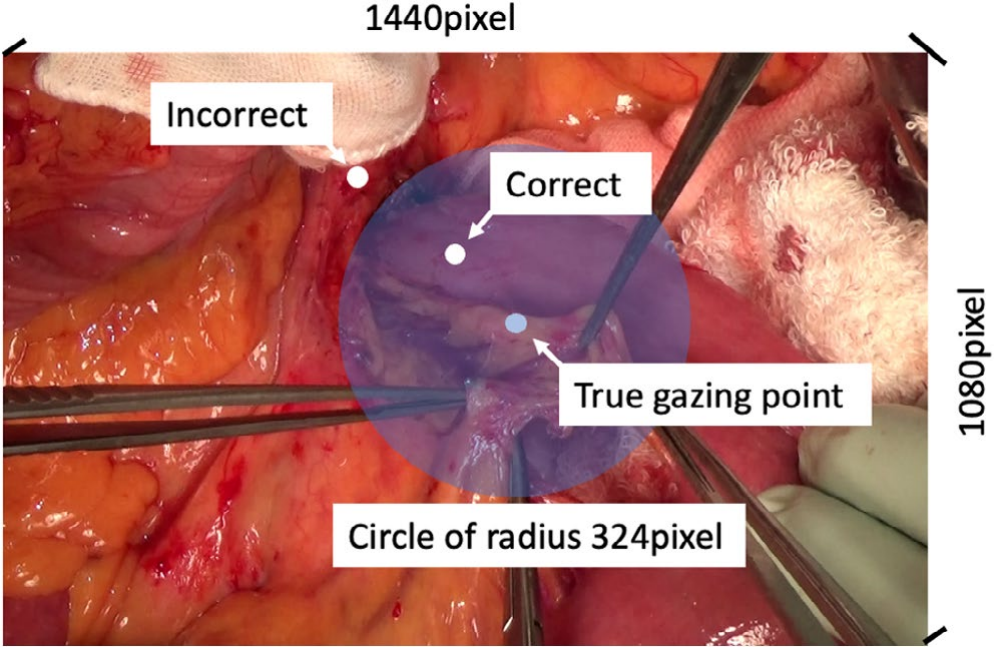
The criteria for the correct estimation of the gazing point. The gazing point detected using the proposed algorithm (white dots) was considered correct when the point was located within a certain pixel of the true gazing point (blue dots). The correct radius for gazing point estimation was set and analyzed at 108, 216, 324, 432, and 540 pixels. Circles with a radius of 324 pixels are indicated by blue circles.

To evaluate the accuracy of our system in detecting gaze points, we compared the gaze points detected by our system with those directly extracted by a surgeon (S.S.). In the test dataset used to validate the entire system, the gazing point of each image was manually determined and annotated by the surgeon (true gazing point). The gazing point detected using our system was considered accurate when it was located within certain pixels (108, 216, 324, 432, or 540 pixels) of the true gazing point on the screen. Pancreaticoduodenectomy cases from 2020 to 2021 had a resolution of 1440 × 1080 pixels (Figure 2), whereas the two extended cholecystectomy and four distal pancreatectomy cases from 2024 and 2025 had a resolution of 1920 × 1080 pixels.

### 2.6 Statistical Analysis

Intersection over Union (IoU) was used to quantify the accuracy of surgical instrument detection. IoU was calculated using the following formula:

$$\mathrm{IoU}=\frac{\text{Area}\;\left(P\right)\cap \text{area}\;\left(\mathrm{GT}\right)}{\text{Area}\;\left(P\right)\cup \text{area}\;\left(\mathrm{GT}\right)}$$

where *P* (prediction) is the detection box generated by the model, and *GT* (ground truth) is the truth box determined by manual annotation. When the IoU of *P* and *GT* exceeded a certain threshold—set at 0.5 (AP50) in this study—the detection was considered correct. Data were analyzed using Microsoft Excel version 16.84. The accuracy of gazing point detection of our system was evaluated using Python 3.11 [14].

## 3 Results

### 3.1 Dataset

Of the five pancreaticoduodenectomy videos used for surgical instrument detection, 291 images from three cases were extracted as training data, and 201 images from two cases were extracted as test data. From the three pancreaticoduodenectomy videos used to validate the entire system for detecting the gaze point, 180 images (60 from each video) were extracted as test data. Similarly, from the six videos (two extended cholecystectomy and four distal pancreatectomy cases) used to validate the entire system for detecting the gazing point, 360 images (60 from each video) were extracted.

### 3.2 Results of CNN‐Based Surgical Instruments Detection

**FIGURE 3:**
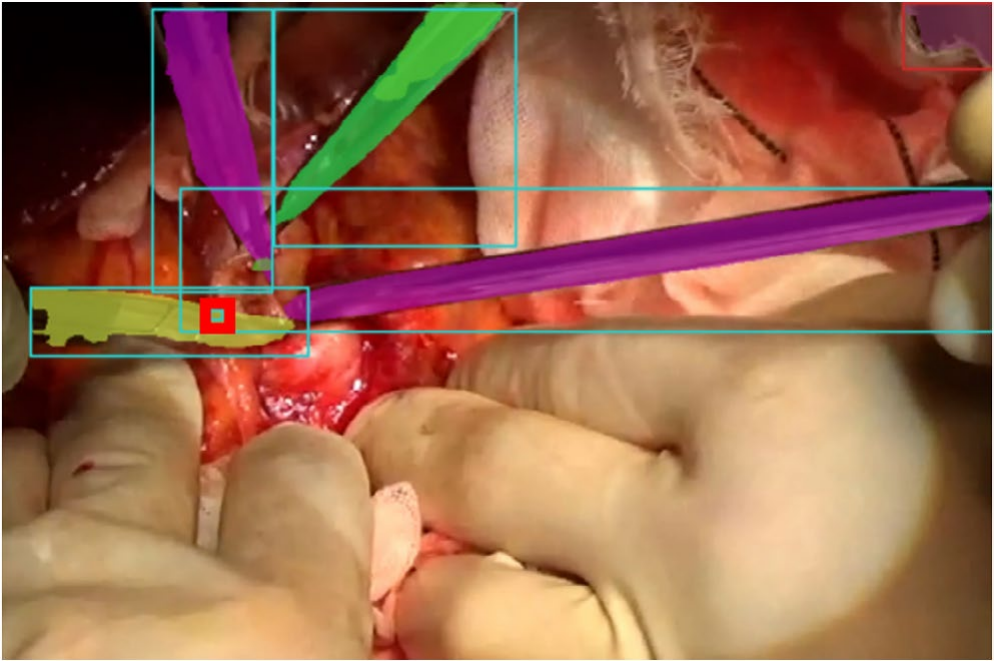
Representative image of surgical instruments detection and gazing point estimation. The yellow, green, and purple areas indicate the detected surgical instruments. Red squares indicate the detected gazing point.

The total cumulative number of surgical instruments was 732 in the training dataset from the 291 images and 506 in the test dataset from the 201 images. The detection rate of the surgical instruments was 60.5% at AP50. Figure 3 presents an example of surgical instrument detection.

### 3.3 Estimation of the Gazing Point Without Time Average

Figure 3 presents an example of gazing point detection. The accuracies for estimating the gazing point without time averaging (Method 1) with radii of 108, 216, 324, 432, and 540 for the correct circle were 0539, 0.828, 0.939, 0.950, and 0.972, respectively. As expected, accuracy increased with larger correct circle sizes. A detection accuracy of 216 or 324 pixels for the gazing point was considered desirable to satisfy both accuracy and clinical utility.

### 3.4 Estimation of the Gazing Point With Time Averaging

**FIGURE 4:**
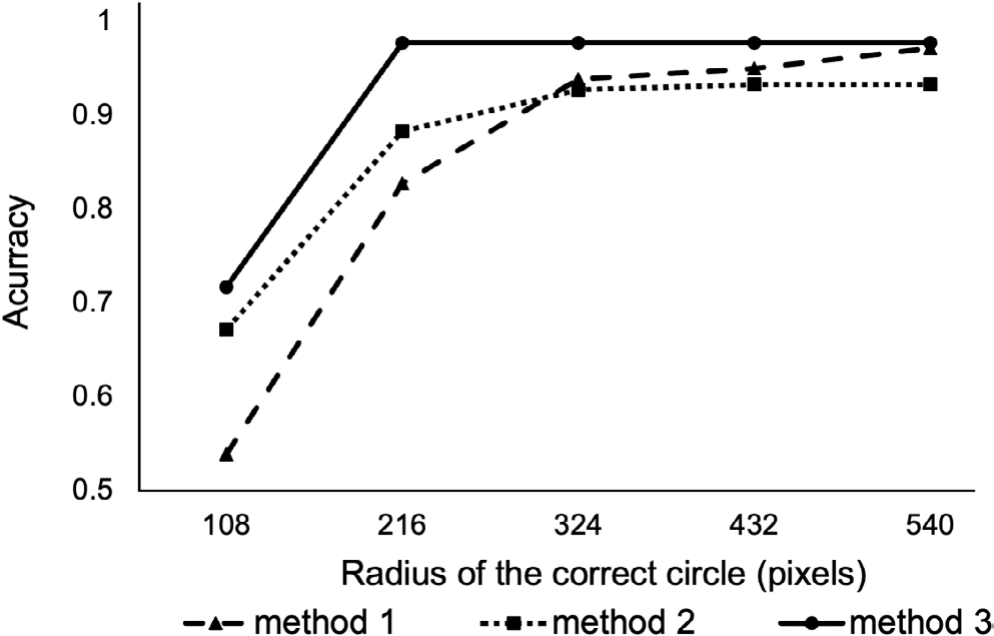
Accuracy of gazing point estimation for each method according to the radius of the correct circle in pancreaticoduodenectomy.

We integrated 4 (1 s) or 20 (5 s) consecutive pictures into one picture, excluding the outlier gazing points, and calculated the center of the estimated gazing points as the time‐averaged gazing point (Figure S2). Figure 4 and Table 1 present the accuracy rate according to the radius of the correct circle stratified by the extent of time averaging. With a radius of 216 pixels for the correct circle, which accounted for 9.42% of the total screen area, the accuracies for Method 1, 2, and 3 were 0.827, 0.833, and 0.978, respectively, and with a radius of 324 pixels, which accounted for 21.2% of the total screen area, the accuracies were 0.939, 0.928, and 0.978, respectively. Method 1 exhibited a good accuracy rate of 0.827 within the 216‐pixel range, and time averaging further improved the accuracy rate. Overall, the performance was better for Method 3 than for Method 1 and 2. The smaller the radius of the correct circle, the greater the benefit of time averaging; however, beyond 324 pixels, time averaging minimally improved the performance. Videos S1 and S2 show the real‐time adaptation of Method 1 and 3 to the surgical video, respectively. In Method 1, the gazing point was detected at every frame (0.25 s); therefore, an excessive fluctuation of the gazing point was observed. In Method 3, incorporating a 5‐s time averaging effectively suppressed gazing point fluctuations.

**TABLE 1:** Accuracy of gazing point estimation for each method according to the radius of the correct circle.

Accuracy	Method 1	Method 2	Method 3
108 pixels			
Case 1	0.667	0.767	0.717
Case 2	0.100	0.367	0.433
Case 3	0.850	0.833	1.000
Average of 3 cases	0.539	0.672	0.717
216 pixels			
Case 1	0.833	0.967	1.00
Case 2	0.633	0.750	0.933
Case 3	0.967	0.933	1.00
Average of 3 cases	0.827	0.833	0.978
324 pixels			
Case 1	0.833	0.983	1.000
Case 2	0.967	0.867	0.933
Case 3	0.967	0.933	1.00
Average of 3 cases	0.939	0.928	0.978

### 3.5 Applicability to Other Hepatobiliary‐Pancreatic Procedures

**FIGURE 5:**
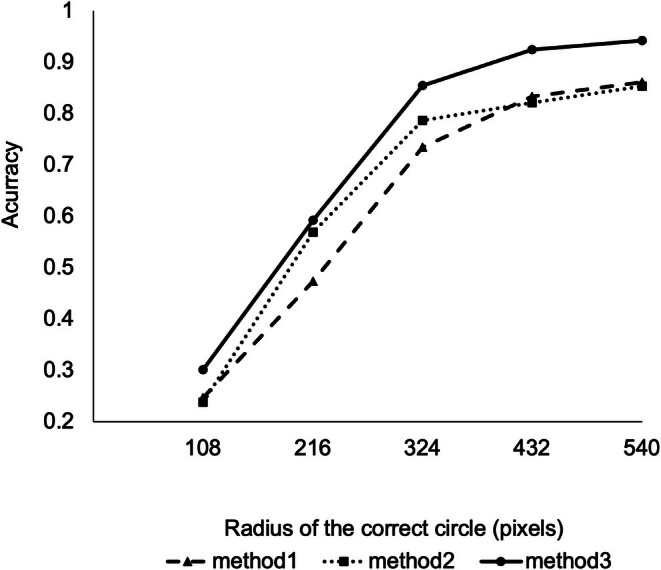
Accuracy of gazing point estimation for each method according to the radius of the correct circle in 2 cases of open extended cholecystectomy and 4 cases of open distal pancreatectomy.

Figure 5 and Table 2 present the accuracy rates according to the radius of the correct circle, stratified by the extent of time averaging. The display resolution was 1920 × 1080 pixels, and a radius of 324 pixels corresponded to 15.8% of the total screen area. At this radius, the accuracy rates for methods 1, 2, and 3 were 0.735, 0.787, and 0.855, respectively.

**TABLE 2:** Average accuracy of gazing point estimation for each method according to the radius of the correct circle for 2 cases of open extended cholecystectomy and 4 cases of open distal pancreatectomy.

Accuracy	Method 1	Method 2	Method 3
108 pixels	0.247	0.237	0.301
216 pixels	0.474	0.568	0.592
324 pixels	0.735	0.787	0.855

## 4 Discussion

In this study, we developed a system that automatically detects the gaze point of a surgeon using surgical video information. Our method is novel in the following respects: First, machine learning was used to detect easily recognizable surgical instruments rather than direct gazing‐point estimation, and the gazing point was calculated from the detected surgical instruments. Instead of directly estimating the gaze point using AI, we adopted an instrument‐based approach. This decision was based on the assumption that the spatial relationship between the position and orientation of surgical instruments and the surgeon's gazing point remains relatively invariant, even under atypical intraoperative conditions such as adhesions, inflammation, bleeding, or anatomical rare variations. This approach has the advantage of identifying the precise point at which surgical manipulation is taking place, regardless of the surrounding anatomical or intraoperative conditions. Subsequently, an algorithm was developed to detect the gazing point from the detected surgical instruments, based on the assumption that the intersection of the instrument axes often corresponds to the gazing point during surgery. The detection rate of surgical instruments was 60.5% at AP50, which was lower than the 85%–92% reported in previous laparoscopic studies that focused on instrument presence classification [4]. It is, however, within the range of 38%–86% reported for instrument detection and localization tasks in laparoscopic settings [4]. This indicates that our detection performance is within the reported range of previous studies. The relatively lower performance in our study can be attributed to the increased complexity of open surgery, where a wider variety of instrument types and shapes are used, instruments are frequently partially obscured by the surgeon's hands, and their positions are far less constrained than in laparoscopic procedures. These factors collectively make instrument recognition more challenging in open surgery. However, the purpose of our system was to estimate the surgeon's gazing point rather than to optimize instrument detection itself, and we adopted the time averaging method to suppress fluctuations in gaze estimation caused by the low detection rate of surgical instruments. Actually, the time averaging improved the accuracy of the gazing point estimation, with the estimation in the 216‐pixel radius range being satisfactory at 0.978 with time averaging every 5 s (Method 3), and we regard the present level of detection accuracy as acceptable for the purpose of this study. The automatic detection of gazing points from images extracted from surgical videos may be useful for increasing the quality of surgical video recordings and improving the accessibility and feasibility of applying AI‐related technologies for surgery in clinical practice. Moreover, by estimating the gaze point from general surgical instrument data rather than procedure‐specific features, our system demonstrated applicability across other hepatobiliary‐pancreatic procedures. Although the accuracy in these procedures was slightly lower than that of pancreatoduodenectomy (85.5% vs. 93.9% with a radius of 324 pixels using method 3), the performance remained clinically acceptable. This decrease may be explained by differences in device usage and video acquisition conditions among procedures.

The surgeon's gazing point is crucial for the clinical application of AI technology when integrating image data obtained from surgical videos. The surgical videos should be captured such that the gazing point is included and centered. Additionally, AI‐related surgical technologies, including the identification of anatomical landmarks [5], recognition of surgical instruments [6], surgical navigation [15], and adverse event detection [16], should be applied relative to the gazing point. However, reports on AI‐based intraoperative gaze point detection remain lacking. Consequently, the clinical application of AI‐related surgical techniques has been challenging, as they require manual camera adjustments. In the present study, we introduce a system that automatically detects the surgeon's gaze point from surgical images using machine learning. Notably, no prior reports have addressed automatic gaze point estimation from surgical images. Our system can be incorporated into existing technologies and can be adapted for future AI‐related technologies regarding surgical images.

Several studies have reported the intraoperative gazing point estimation using different techniques. For example, Lee et al. reported gazing point recordings using the EyeLink II Eye Tracking System (SR Research Ltd., Ontario, CA, USA) during laparotomy [17]. Rana et al. reported a Tobii X50 Eye Tracker (Tobii AB, Stockholm, Norway) for measuring gaze points during laparoscopic surgery [18]. These devices have demonstrated a significant difference in gazing points between skilled and novice surgeons during surgery. Both EyeLink II Eye‐Tracking System and Tobii X50 Eye Tracker automatically detect intraoperative gazing points; however, they require specialized and expensive equipment. Meanwhile, our system, developed using machine learning and mathematical algorithms, automatically detects intraoperative gaze points from surgical images. The results indicated that the detected gazing point was within a 216‐pixel radius of the true gazing point, achieving an accuracy of 0.978. This radius accounted for 9.42% of the total screen area, representing a sufficiently limited and practical range for clinical applications. Importantly, in our system, the gazing point detection feature has already been developed as an application, requiring only an ordinary laptop computer to automatically detect the gazing point from surgical images, making it more feasible than previously reported methods.

Detection of the intraoperative gazing point using our simple system offers key advantages. In laparotomy, integrating our system with a video recorder allows for video recording with automatic tracking and zooming based on the surgeon's gaze point. Video recording is necessary for medical records, ensuring patient safety [19], providing materials for research and education [20], and sharing information with other professionals such as anesthesiologists and co‐medics [21]. Conventional video recording of open surgery relies on personnel to manually or remotely adjust the camera when the recording deviates from the surgical operation site. However, automating camera operation using our system reduces the need for additional personnel [22]. In laparoscopic surgery, devices capable of holding and controlling an endoscopic camera via a controller or voice commands are already in use [23]. To automate camera operation in laparoscopic surgery, Nishikawa et al. reported an automatic laparoscope positioning system that constantly measures the three‐dimensional positions of the surgical instruments used by the surgeon and the laparoscopic camera held by the device, determining camera movements based on this information [24]. Integrating our system could enable automatic camera operation based on the surgeon's gaze point, eliminating the need for manual instructions for manipulating the endoscopic camera, thereby allowing the surgeon to focus more on the surgery. This could also reduce the need for human resources in surgery, benefiting the surgical fields facing a shortage of surgeons.

This study also has some limitations. First, it was conducted at a single institution using a single type of procedure as training data, which limited the types of surgical instruments appearing in the surgical images. Our system has not been tested for reproducibility at other institutions or in procedures outside hepatobiliary‐pancreatic surgery. However, since surgical instruments and their use in open hepatobiliary‐pancreatic surgery are common across various procedures, our system could be applicable to open abdominal surgery in other fields, such as gastroenterology, urology, obstetrics, and gynecology. Second, at least two surgical instruments are required to estimate the gazing point using our system. Therefore, it may be difficult to estimate the gazing point in procedures that directly use the surgeon's hands rather than surgical instruments. Third, an experienced surgeon manually annotated the true gaze point to validate our system. Although the surgeon sets the gazing point based on information in the image, independent of the position of the surgical instruments, it may be difficult to define a correct and objective gazing point for everyone. As prior research in this field remains limited, no established validation protocol for gaze point assessment currently exists. Although further standardization is warranted, the present approach may serve as a potential basis for future methodological development in gaze research. Finally, this study focused on the detection of gaze points in open surgery and not on gaze point estimation in minimally invasive surgery. In laparoscopic surgery, gaze dynamics might correlate with the scopist's viewpoint rather than the operator's, and therefore, it is necessary to verify whether the insight that the gazing point corresponds to the intersection of the forceps axes remains valid in the laparoscopic surgical environment. Future studies are warranted to verify whether our system is also effective in estimating the gazing point in minimally invasive surgery.

## 5 Conclusion

Using AI and the original algorithm, our system successfully detected the gazing point from images extracted from surgical videos. This system exhibits the potential to enhance the quality of surgical video recordings and improve the accessibility and feasibility of applying AI‐related technologies for surgery in clinical practice.

## Funding

The authors have nothing to report.

## Conflicts of Interest

The authors declare no conflicts of interest.

## Supporting information


**Figure S1:** Algorithm to estimate the axis of surgical instruments. (a) A red rectangle that contains the instrument is a detection box. The blue area is a detection area of the target instrument. The center of the instrument was calculated from detection area information.
**Figure S2:** Time averaging of the gazing points over four frames (1 s). The yellow and blue dots indicate the estimated gaze points. The blue dot represents an outlier and is excluded. The red point represents the center of gravity of the three yellow dots, signifying the estimated gazing point after following time averaging over the four frames (1 s).

**Video S1:** Real‐time adaptation of Method 1 to a pancreaticoduodenectomy video. In Method 1, the gazing point was detected at every frame (0.25‐s interval), which resulted in excessive fluctuation of the gazing point.

**Video S2:** Real‐time adaptation of Method 3 to a pancreaticoduodenectomy video. In Method 3, incorporating a 5‐s time‐averaging process effectively suppressed the fluctuation of the gazing point.

## Data Availability

The data that support the findings of this study are available on request from the corresponding author. The data are not publicly available due to privacy or ethical restrictions.
