# Supplementary material for: Automatic and Real‐Time Surgeon's Gazing Point Detection From Surgical Videos Using Machine Learning and Mathematical Algorithm
Source: J Hepatobiliary Pancreat Sci. 2025 Dec 19;33(3):161–7. doi: 10.1002/jhbp.70052 (PMC12993703; doi:10.1002/jhbp.70052)
Supplement: Supplementary file 1 — Figure S1: Algorithm to estimate the axis of surgical instruments. (a) A red rectangle that contains the instrument is a detection box. The blue area is a detection area of the target instrument. The center of the instrument was calculated from detection area information. Figure S2: Time averaging of the gazing points over four frames (1 s). The yellow and blue dots indicate the estimated gaze points. The blue dot represents an outlier and is excluded. The red point represents the center of gravity of the three yellow dots, signifying the estimated gazing point after following time averaging over the four frames (1 s). [file JHBP-33-161-s002.zip › jhbp70052-sup-0003-FigureS1-S2@Figure legendsSuppInfo 20250714.docx]

**Supplementary data**

Figure S1. Algorithm to estimate the axis of surgical instruments. (a) A red rectangle that contains the instrument is a detection box. The blue area is a detection area of the target instrument. The center of the instrument was calculated from detection area information.

(b) A circle with the radius of

$$\frac{\sqrt{{height}^{2}+{width}^{2}}}{2}\times\frac{3}{4}$$

was placed at eight points positioned at height/4 and width/4 away from the center of gravity. Of the circles, the center of interest is the circle that shares most of the detection area (dark red circle). The three circles on the opposite side of the circle of interest are opposite circles (light red circles). The center of the detection area contained within the circle of interest is named α. The centers of gravity of the detection area contained within each of the opposite circles are named β1, β2, and β3. (c) γ is the center of gravity of β1, β2, and β3. The line passing through the points α and γ represents the axis of the instrument.

Figure S2. Time averaging of the gazing points over four frames (1 s). The yellow and blue dots indicate the estimated gaze points. The blue dot represents an outlier and is excluded. The red point represents the center of gravity of the three yellow dots, signifying the estimated gazing point after following time averaging over the four frames (1 s).
