# Supplementary figures and images for: Automatic and Real‐Time Surgeon's Gazing Point Detection From Surgical Videos Using Machine Learning and Mathematical Algorithm
Source: J Hepatobiliary Pancreat Sci. 2025 Dec 19;33(3):161–7. doi: 10.1002/jhbp.70052 (PMC12993703; doi:10.1002/jhbp.70052)

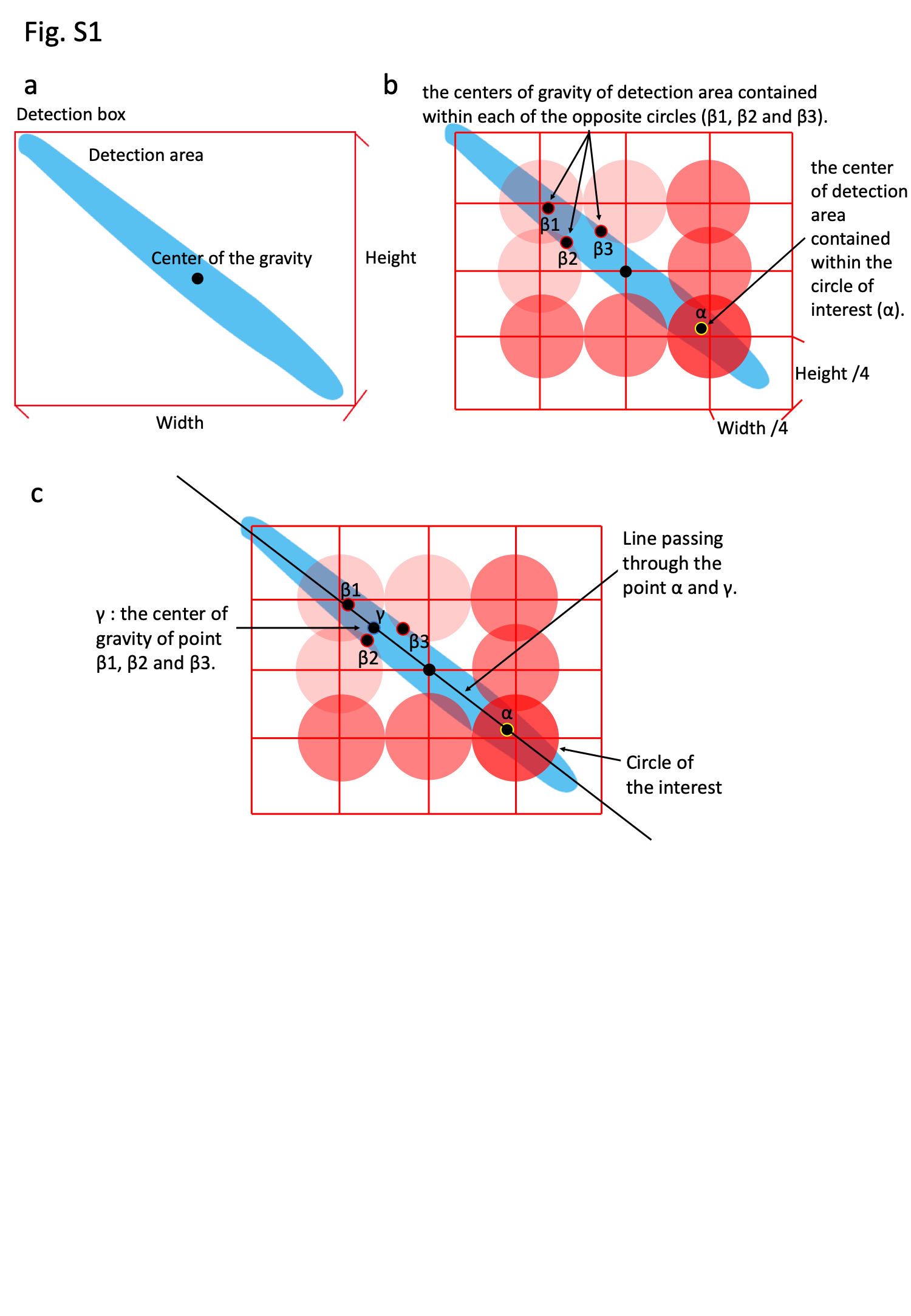

Supplement: Supplementary file 1 — Figure S1: Algorithm to estimate the axis of surgical instruments. (a) A red rectangle that contains the instrument is a detection box. The blue area is a detection area of the target instrument. The center of the instrument was calculated from detection area information. Figure S2: Time averaging of the gazing points over four frames (1 s). The yellow and blue dots indicate the estimated gaze points. The blue dot represents an outlier and is excluded. The red point represents the center of gravity of the three yellow dots, signifying the estimated gazing point after following time averaging over the four frames (1 s). [file JHBP-33-161-s002.zip › 1_FigureS1Supplnfo.tiff]

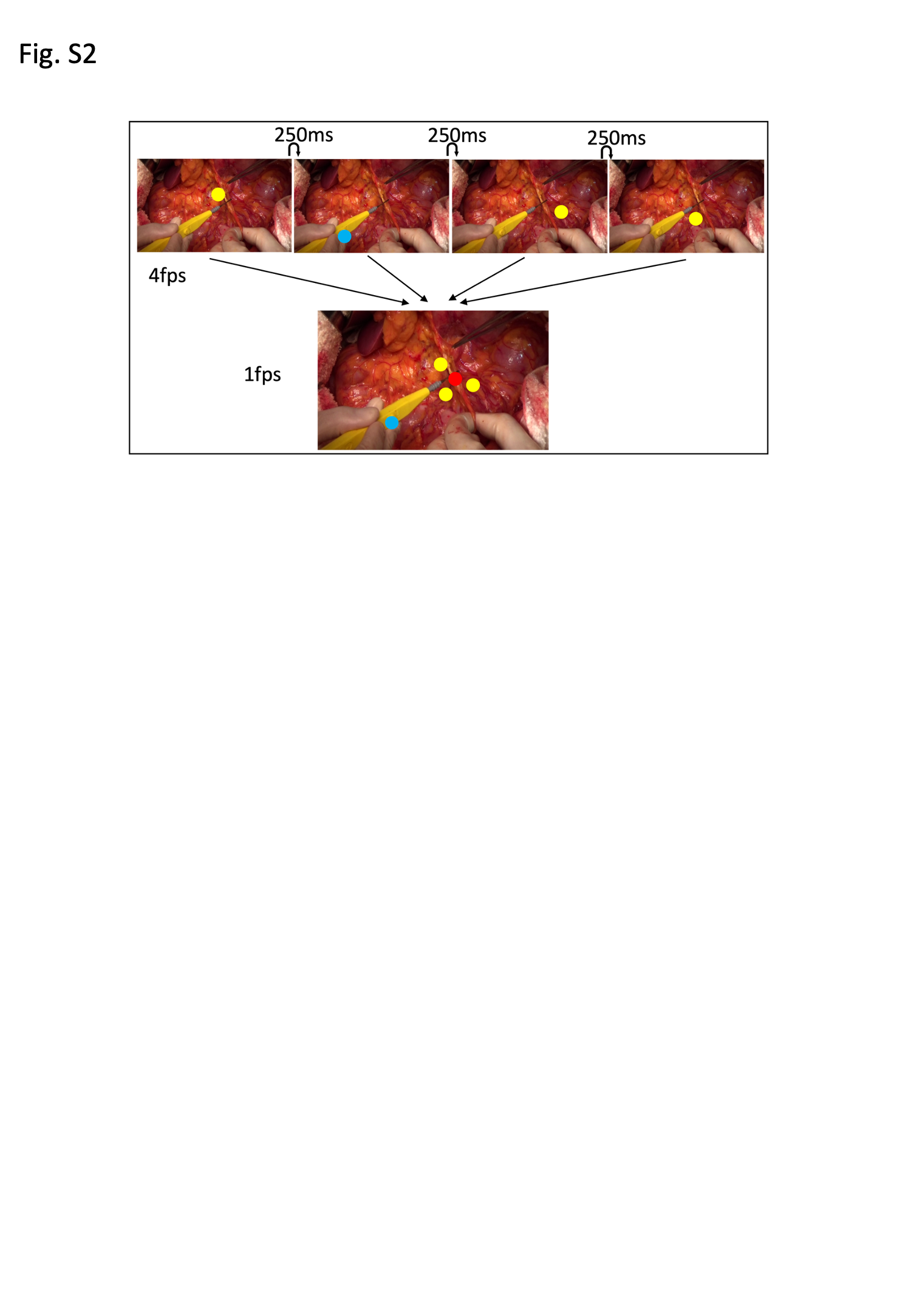

Supplement: Supplementary file 1 — Figure S1: Algorithm to estimate the axis of surgical instruments. (a) A red rectangle that contains the instrument is a detection box. The blue area is a detection area of the target instrument. The center of the instrument was calculated from detection area information. Figure S2: Time averaging of the gazing points over four frames (1 s). The yellow and blue dots indicate the estimated gaze points. The blue dot represents an outlier and is excluded. The red point represents the center of gravity of the three yellow dots, signifying the estimated gazing point after following time averaging over the four frames (1 s). [file JHBP-33-161-s002.zip › 1_FigureS2SuppInfo.tiff]
